# Regulatory Role of miR-203 in Occurrence and Progression of Kazakh Esophageal squamous cell carcinoma

**DOI:** 10.1038/srep23780

**Published:** 2016-03-31

**Authors:** Jian Ming Hu, Ai Min Chang, Yun Zhao Chen, Xiang Lin Yuan, Feng Li

**Affiliations:** 1 Department of Pathology and Key Laboratory of Xinjiang Endemic and Ethnic Diseases (Ministry of Education), Shihezi University School of Medicine, Shihezi 832003, China; 2 Department of Pathology, Beijing Chaoyang Hospital, Capital Medical University, Beijing, 100020, China; 3 Department of Oncology, Tongji Hospital, Tongji Medical College, Huazhong University of Science and Technology, Wuhan 430030, China; 4 Department of Pathology, Wusu people’s hospital, Wusu, Xinjiang, 833000, China

## Abstract

Esophageal carcinoma is one of the most common malignant tumors and the Kazakh national minority (ethnic) in Xinjiang (northwest of China) has been reported to be one of the highest incidence of Esophageal squamous cell carcinoma (ESCC) in the world. MicroRNA-203 (miR-203) was described as a tumor-suppressive miRNA in several cancers, but little study about the role of miR-203 in Kazakh ESCC. Therefore, we aimed to investigate the role of miR-203 in the occurrence and progression of Kazakh ESCC. Quantitative real-time polymerase chain reaction (qRT-PCR) was used to detect miR-203 expression, and immunohistochemistry (IHC) was used to examine P63 expression. The expression level of miR-203 in ESCC was significantly lower than that of cancer adjacent normal (CAN) samples (P < 0.05). Whereas the expression level of P63 in ESCC was significantly higher than that of CAN samples (P < 0.05), an inverse association between the expression of P63 and miR-203 was found but was not statistically significant (P > 0.05). These findings suggest that miR-203 is a tumor suppressor gene that plays an important role in inhibiting the occurrence of Kazakh ESCC in Xinjiang, China.

Esophageal carcinoma is one of the most common malignant tumors in the world. Based on its etiological and pathological characteristics, it is divided into two main forms: esophageal squamous cell carcinoma (ESCC) and esophageal adenocarcinoma. More than 90% of esophageal carcinoma are classified as ESCC in China^1^. The incidence rate varies in different physiographical regions, nations and races. China has a high incidence of ESCC with a high mortality rate for these patients^2^. The Kazakh national minority (ethnic) in Xinjiang (northwest of China) has been reported to exhibit the highest incidence of ESCC^3^. The occurrence of ESCC is a complex process involving multiple factors, stages, and interactions^4^. In our previous study, we observed the abnormal expression and methylation of some encoding genes that may increase the risk of Kazakh ESCC^5^,^6^,^7^. Recently, non-coding genes have gradually become the hotspot for cancer research, in particular, increasing attention has been placed on micro RNAs (miRNAs)^8^.

MiRNAs are small, non-coding RNA molecules that regulate the expression of protein-coding genes via base pairing to the 3′-untranslated region (3′UTR), which affects gene silencing via both translational inhibition and mRNA degradation. Some miRNAs can regulate lots of distinct mRNAs^9^. The dysregulation of miRNA expression has been identified in various cancers and suggests that miRNAs can function as classical oncogenes or tumor suppressor genes^10^.

MiRNA-203 (miR-203) is expressed specifically in the suprabasal layers of stratified epithelia, which is an antiproliferative miRNA involved in squamous epithelium differentiation that targets the 3′-UTR of the transcription factor p63 family^11^,^12^. In addition, miR-203 has been shown to act as a tumor-suppressive miRNA, and the down-regulation of miR-203 expression is described in several types of cancer, including lung cancer^13^, hepatocellular carcinoma^14^, pancreatic cancer^15^ and laryngeal squamous cell carcinoma^16^. Although the aberrant expression of miR-203 has also been reported in ESCC^17^,^18^, the expression of miR-203 in Kazakh ESCC is unclear. Furthermore, most studies investigating the role of miR-203 in ESCC have been conducted *in vitro* and little in *ex vivo*. In this study, we aimed to investigate whether the aberrant expression of miR-203 exists in Kazakh ESCC. Additionally, we assessed whether the regulation of miR-203 to P63 is associated with the occurrence and progression of Kazakh ESCC in Xinjiang, China.

## Results

### MiR-203 expression and its relationship with ESCC clinicopathological parameters

Using NanoDrop to confirm RNA quality, qRT-PCR was used to evaluate miR-203 expression in 94 cases of Kazakh ESCC and 72 cases of CAN. The expression level of miR-203 in Kazakh ESCC was 0.210 ± 0.341, which was significantly lower than that of CAN 0.803 ± 1.332 (P < 0.05) (Table 1).

To assess the meaningful expression of miR-203 in the progression of Kazakh ESCC, we analyzed the relationship between miR-203 expression and clinicopathological factors. The expression of miR-203 was lower in lymph node metastasis, as compared to that in the absence of metastasis. However, this difference was not statistically significant (P = 0.089). No significant differences were noted in the other parameters, such as age, gender, histological differentiation and clinical stages (P > 0.05) (Table 2).

### P63 expression in Kazakh ESCC and CAN tissues and correlations between P63 expression and ESCC clinicopathological parameters

**Figure 1:**
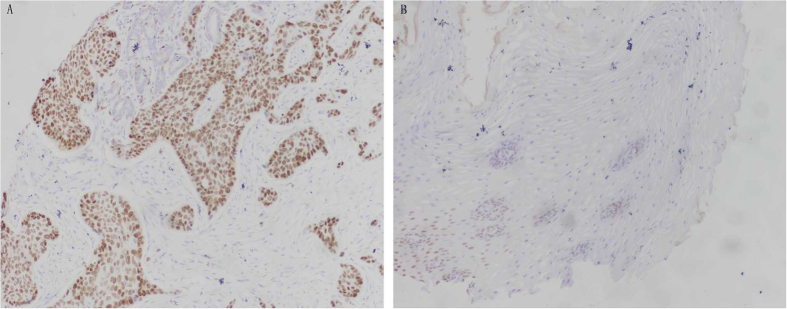
Immunohistochemical staining of P63 in Kazakh ESCC and CAN tissues. (**A**) Esophageal squamous cell carcinoma(ESCC) tissue. Note strong staining of P63 (3+) in squamous epithelial cancer cells (original magnification ×200). (**B**) Cancer adjacent normal esophageal tissue(CAN). Note the weak positive staining (+)of P63 in squamous epithelial cells (original magnification ×200).

The nuclei of tumor cells exhibited P63-positive staining (Fig. 1). P63 protein expression was observed in 71 (83.5%) of 85 ESCC tissue samples; of these, 54 (63.5%) had moderate or strong expression (2+/3+), and 17 (20.0%) had weak (1+) expression. Only 14 cases of ESCC (16.5%) were negative for P63. Although P63 expression was observed in 22 (59.4%) of 37 CAN tissue samples, only 8 (21.6%) cases exhibited moderate or strong expression (2+/3+). The expression of P63 protein in ESCC was higher than that of CAN tissues, and the difference was especially prominent with regard to moderate or strong (2+/3+) staining (P < 0.05) (Table 3).

To assess the role of P63 expression in Kazakh ESCC, we examined possible correlations between P63 expression and various clinicopathological parameters, including age, gender, histological grade, nodal status and clinical stages. The decreased expression of P63 was significantly correlated with poorly differentiated ESCC, as compared to the correlation of P63 with well-differentiated ESCC (P < 0.05). Interestingly, P63 expression was increased in ESCC compared to that of CAN, while as well-differentiated ESCC progressed to moderately and poorly differentiated ESCC, the expression of P63 decreased. No significant correlations were found between P63 expression and other parameters (P > 0.05) (Table 4).

### Correlation between the expression of miR-203 and P63 in ESCC

Previous studies have shown that P63 is a target gene of miR-203. The regulation of P63 expression by miR-203 has been reported in ESCC *in vitro* but little *in human ex-vivo*. Therefore, we evaluated the regulated function of miR-203 to the expression of P63 in Kazakh ESCC tissues. We found P63 expression increased gradually as miR-203 expression decreased. However, this negative regulation of P63 by miR-203 had no statistical significance (*P* > 0.05) (Table 5).

## Discussion

MiR-203 has been shown the function of inducing squamous differentiation of epidermal cells, and inhibiting Cell growth in various cancers, even most study found *in vitro* that miR-203 inhibits the progression of cancer by regulating some target genes^19^,^20^,^21^. However, few studies about the role of miR-203 in human *ex-vivo* esophageal squamous cell carcinoma(ESCC) occurrence and progression, and some studies even contradicted with variable results depending on the different ethnic groups. In this study, we used qRT-PCR to detect the expression of miR-203 in Kazakh ESCC and cancer normal adjacent mucosa (CAN), found that the expression of miR-203 was clearly decreased in ESCC compared to that in the CAN. These results are similar to those reported in ESCC cell lines^17^,^18^, and similar with Hezova R^22^ and Slaby O^23^ in Czech republic esophageal adenocarcinoma (EAC) tissues study. However, Stánitz É^24^ got contrary results in their study. They found miR-203 was over-expressed in ESCC compared with normal tissues. Different ethnic groups have different genetic background that may have been cited as potential causes of inconsistency. In ESCC cell lines, miR-203 has been shown to suppress tumors and inhibit the growth, invasion and metastasis of tumor cells. We also assessed whether miR-203 has similar functions in Kazakh ESCC patients. The analysis of clinicopathological factors demonstrates that miR-203 expression was lower in lymph node metastasis than in non-metastatic conditions. However, this decrease of expression was not statistically significant, and no correlations between other clinical parameters and miR-203 expression were found.

MiR-203 has also been shown to inhibit ESCC cells growth and invasion through the suppression of P63^17^. P63 is a member of the P53 gene family that encodes several protein isoforms. It is amplified in a significant proportion of squamous cell carcinoma, including squamous cell carcinoma of the lung^25^, head and neck^26^ and cervix^27^. In this study, we found that the expression of P63 in Kazakh ESCC was higher than that of CAN samples, and ESCC tissues exhibited more instances of strong P63-positive expression. The results is similar with some early studies^28^,^29^. However, Cao^30^ found the expression of p63 in ESCC samples was lower than that in normal mucosa. To explain these differences in the reports, sampling methods, geographic area, ethnic factors and antibody choosing (encodes different protein isoforms) have been cited as potential causes of inconsistency. To assess the role of P63 in progression of Kazakh ESCC, we examined possible correlations between P63 expression and clinicopathological parameters, and found the expression level of P63 was significantly decreased with poorly differentiated ESCCs as compared with well-differentiated ESCCs, however, the expression of P63 was higher in ESCC compared with that of CAN samples. Which means the expression of P63 increased in the progression of ESCC carcinogenesis, while the gradually decreased in the evolution of ESCC from well to poorly differentiated, the results is similar with Wang^31^ and Quade^32^ in cervical cancer study, they found patients with well-differentiated cervical cancer exhibit strong P63-positive expression, while P63 expression became gradually undetectable as the cancer progresses to the undifferentiated state. This may suggest that P63 plays a promoting role in carcinogenesis but plays an inhibiting role in tumor differentiation.

P63 is an essential regulator of stem-cell maintenance in stratified epithelial tissues^33^. The 3′-UTR contains a binding site for miR-203, which can repress the expression of P63 and inhibit cell proliferation in human skin epithelial precursor cells^12^. To evaluate whether miR-203 negatively regulates P63 in Kazakh ESCC patients, we analyzed the correlation of miR-203 with the expression of P63. Following the decreased expression of miR-203, the expression of P63 increased gradually. Although there is a reverse regulation from miR-203 to P63, the regulation function has no statistically significant. Therefore, other factors may play more important roles in the regulation of P63 in Kazakh ESCC.

A limitation in our works is the loss of other epidemiology information, such as alcohol use, smoking status and eating habits, which may help us to assess the interaction between miR-203 and other environment factors in esophageal carcinoma. Another limitation is the lack of follow-up data, which would help us analysis some prognostic factors, and might further explain the meaningful of miR-203 in Kazakh ESCC prognostic assessment.

## Materials and Methods

### Ethics Statement

All participating were recruited from the Yili Friendship Hospital in Xinjiang, China. Each participant provided written informed consent before enrollment in this study, and the protocols were approved by the institutional ethics committee at Yili Friendship Hospital in accordance with the ethical guidelines of the Helsinki Declaration. The methods were carried out in accordance with the approved guidelines.

### Study population

Ninety-four Kazakh specimens were collected between 2005 and 2011. The patients were 43–75 years old (49 men and 45 women) and had been diagnosed with ESCC. These patients did not receive radiotherapy or chemotherapy before surgery. Seventy-two specimens were collected from cancer adjacent normal (CAN) tissues as controls. The CAN group participants were 45–73 years old (42 men and 30 women). All specimens (94 ESCC and 72 CAN) were used for miR-203 detection, and 85 ESCC and 37 CAN specimens were used for P63 detection.

All ESCC specimens were obtained after surgery and were embedded in paraffin. Afterwards, they were sectioned into 5-μm slices and subjected to conventional hematoxylin and eosin staining. The diagnosis of ESCC was confirmed by two pathologists, according to the World Health Organization histological tumor classification criteria^34^. There were 21 cases of well-differentiated ESCC, 56 cases of moderately differentiated ESCC, and 17 cases of poorly differentiated ESCC. Of which 51 patients had lymph node metastasis, 43 without lymph node metastasis, 63 cases in TNM stage I–II, and 23 cases in stage III–IV. CAN specimens, which were sampled at more than 5 cm away from the cancer region, were confirmed to be free of cancer tissue.

### Immunohistochemical analysis

The paraffin-embedded tissue samples were cut into 4-μm-thick sections and mounted on polylysine-coated slides. The samples were dewaxed in xylene and rehydrated using a graded series of ethanol solutions. After deparaffinization, endogenous peroxidase activity was blocked by incubation in a 3% peroxide-methanol solution at room temperature for 10 minutes, and then antigen retrieval was performed at 100 °C in an autoclave for 7 minutes. Samples were incubated at room temperature for 30 minutes. Afterwards, sections were washed with phosphate-buffered saline (PBS) 3 times for 5 minutes each time. They were then incubated with the primary anti-P63 monoclonal antibody (1:100; DAKO, Glostrup, Denmark) overnight at 4 °C. Next, a thorough washing with PBS was performed, and the binding of the primary antibody was visualized using a DAKO EnVision kit (DAKO, Glostrup, Denmark), according to the manufacturer’s instructions. Finally, sections were faintly counterstained with hematoxylin and mounted with glycerol gelatin. A positive control (P63-positive sample) and a negative control (PBS) were included in these experiments. All sections were analyzed under a light microscope by two experienced pathologists. Results were scored as positive or negative by the percentage and intensity of positive cells. The percentage of positive cells was scored as 0 in the absence of staining, 1 for less than 25% stained cells, 2 for 25–50% stained cells, and 3 for more than 50% stained cells. The intensity of staining was scored as 0, 1, 2, or 3 in reference to absent, weak, clear, or strong expression, respectively. The staining results were divided into 3 categories based on the sum of both scores: 0 was negative (−), 1–2 was weak positive (1+), 3–4 was moderately positive (2+), and 5–6 was strong positive (3+).

### Quantitative real-time polymerase chain reaction (qRT-PCR)

RNA was isolated using the RNeasy Mini Kit (Qiagen, Hilden, Germany), according to the manufacturer’s specifications. Complementary DNA (cDNA) was generated with the High-Capacity cDNA Reverse Transcription kit (Qiagen, Hilden, Germany). Quantitative RT-PCR was performed using SYBR green PCR Master Mix (Qiagen, Hilden, Germany) containing ROX as a reference dye. Each qRT-PCR assay was conducted in triplicate using cDNA derived from 50 ng total RNA (miR-203-positive or miR-203-negative samples). The ratios of miRNA amounts were compared among samples. Triplicate Ct values were averaged, and the relative expression levels of ESCC were determined as 2^−ΔCt^ (ΔCt = Ct of miR-203 in ESCC tissues − Ct of U6 small nuclear RNA (*RNU6*) in ESCC tissues), and the relative expression levels of CAN were determined as 2^−ΔCt^ (ΔCt = Ct of miR-203 in CAN tissues − Ct of U6 small nuclear RNA (*RNU6*) in CAN tissues).

### Statistical analysis

The SPSS version 13.0 software was employed for all statistical analyses. Correlations between P63 staining were calculated using the Pearson χ^2^ test. Two-sample t-tests were conducted to compare miR-203 expression between ESCC and normal tissues. Spearman correlation was used to evaluate the correlations between the expression levels of P63 and miR-203. *P* values were calculated by the Epi-Info program, and *P* values less than 0.05 were considered significant.

## Additional Information

**How to cite this article**: Hu, J. M. *et al.* Regulatory Role of miR-203 in Occurrence and Progression of Kazakh Esophageal squamous cell carcinoma. *Sci. Rep.* **6**, 23780; doi: 10.1038/srep23780 (2016).

## Figures and Tables

**Table 1:** The Expression of miR-203 gene in Kazakh’ESCCs and CANs tissues.

Groups	Cases (N)	miR-203 expression quantity	T	*P*
ESCCs	94	0.210 ± 0.341	−4.148	<0.001
CANs	72	0.803 ± 1.332		

**Table 2:** Correlation between clinicopathologic data and miRNA-203 expression in Kazakh ESCCs.

clinicopathological features	Cases	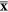 ± S	T/F	*P*
gender				
Male	49	0.249 ± 0.393	1.682	0.097
Female	45	0.180 ± 0.296		
Age (y)				
≤Median (60y)	51	0.288 ± 0.484	1.343	0.183
>Median	43	0.180 ± 0.227		
Histologic grade				
Well	21	0.196 ± 0.267	0.574^*^	0.565
Moderate	56	0.226 ± 0.379		
Poor	17	0.130 ± 0.130		
Nodal status				
pN+	51	0.155 ± 0.343	−1.720	0.089
pN−	43	0.275 ± 0.331		
TNM stage^┼^				
I–II	63	0.215 ± 0.293	0.186	0.852
III–IV	31	0.201 ± 0.427		

*F value. ^┼^TNM stage: the clinicopathologic stage.

**Table 3:** The expression of P63 in Kazakh’s ESCC and the CAN.

P63	ESCC (n = 85)	CAN (n = 37)	X^2^	*P*	*OR(95%)*
−	14	15			
+	17	14	0.062	0.803	1.307(0.471–3.591)
2+/3+	54	8	13.778	0.000^*^	7.233(2.556–20.463)

*P < 0.05.

**Table 4:** Correlation between clinicopathologic data and P63 expression in the Kazakh ESCC.

Clinicopathologic Parameter	No. of Cases	Expression of P63		X^2^	*P*
		Positive n (%)	Negative n (%)		
Age (y)					
≤Median (60y)	45	39(86.7%)	6(13.3%)		
>Median	40	32(80.0%)	8(20.0%)	0.739	0.691
Gender					
M	56	48(85.7%)	8(14.3%)		
F	29	23(79.3%)	6(20.7%)	0.653	0.721
Histologic grade					
Well	17	15(88.2%)	2(11.8%)		
Moderate	56	47(83.9%)	9(16.1%)	0.189	0.664
poor	12	5(41.7%)	7(58.3%)	7.128	0.008^*^
Nodal status					
pN^+^	41	35(85.3%)	6(14.7%)		
PN^−^	44	37(84.1%)	7(15.9%)	0.055	0.973
TNM stage					
I–II	62	53(85.5%)	9(14.5%)		
III–IV	23	19(82.6%)	4(17.4%)	0.107	0.948

*P < 0.05.

**Table 5:** Correlation between the expression of miR-203 and P63 in ESCCs.

P63 expression	No. of Cases	miR-203 expression	*R*	*P*
−	12	0.900 ± 0.000	−0.120	0.670
+	15	0.627 ± 0.876	−0.209	0.895
2+/3+	22	0.317 ± 0.590	−0.354	0.387

## References

[b1] Parkin D. M., Bray F., Ferlay J. & Pisani P. Global cancer statistics, 2002. CA Cancer J Clin 55, 74–108 (2005). 15761078 10.3322/canjclin.55.2.74

[b2] Chen W. *et al.* Esophageal cancer incidence and mortality in China, 2009. J Thorac Dis 5, 19–26 (2013). 23372946 10.3978/j.issn.2072-1439.2013.01.04 PMC3547988

[b3] Ya M. Z. The distribution of esophageal cancer in Xinjiang. J Xinjiang Med Univ (China) 11, 139–144 (1988).

[b4] Cheung W. Y. & Liu G. Genetic variations in esophageal cancer risk and prognosis. Gastroenterol Clin North Am 38, 75–91 (2009). 19327568 10.1016/j.gtc.2009.01.009

[b5] Hu J. M. *et al.* HLA-DRB1 and HLA-DQB1 methylation changes promote the occurrence and progression of Kazakh ESCC. Epigenetics 9, 1366–1373 (2014). 25437052 10.4161/15592294.2014.969625 PMC4623353

[b6] Hu J. M. *et al.* HLA-DRB1*1501 and HLA-DQB1*0301 alleles are positively associated with HPV16 infection-related Kazakh esophageal squamous cell carcinoma in Xinjiang China. CII 61, 2135–2141 (2012). 22588649 10.1007/s00262-012-1281-x PMC11029737

[b7] Hu J. *et al.* Overexpression of HLA-G Is positively associated with Kazakh esophageal squamous cell carcinoma in Xinjiang, China. Viral Immunol 26, 180–184 (2013). 23772974 10.1089/vim.2012.0085

[b8] Alvarez-Garcia I. & Miska E. A. MicroRNA functions in animal development and human disease. Development 132, 4653–4662 (2005). 16224045 10.1242/dev.02073

[b9] Erson A. E. & Petty E. M. MicroRNAs in development and disease. Clin Genet 74, 296–306 (2008). 18713256 10.1111/j.1399-0004.2008.01076.x

[b10] Esquela-Kerscher A. & Slack F. J. Oncomirs - microRNAs with a role in cancer. Nat Rev Cancer 6, 259–269 (2006). 16557279 10.1038/nrc1840

[b11] Lena A. M. *et al.* miR-203 represses ‘stemness’ by repressing DeltaNp63. Cell Death Differ 15, 1187–1195 (2008). 18483491 10.1038/cdd.2008.69

[b12] Yi R., Poy M. N., Stoffel M. & Fuchs E. A skin microRNA promotes differentiation by repressing ‘stemness’. Nature 452, 225–229 (2008). 18311128 10.1038/nature06642 PMC4346711

[b13] Wang C. *et al.* miR-203 inhibits cell proliferation and migration of lung cancer cells by targeting PKCalpha. PloS one 8, e73985 (2013). 24040137 10.1371/journal.pone.0073985 PMC3769394

[b14] Furuta M. *et al.* miR-124 and miR-203 are epigenetically silenced tumor-suppressive microRNAs in hepatocellular carcinoma. Carcinogenesis 31, 766–776 (2010). 19843643 10.1093/carcin/bgp250

[b15] Ikenaga N. *et al.* MicroRNA-203 expression as a new prognostic marker of pancreatic adenocarcinoma. Ann Surg Oncol 17, 3120–3128 (2010). 20652642 10.1245/s10434-010-1188-8

[b16] Tian L. *et al.* MiR-203 is downregulated in laryngeal squamous cell carcinoma and can suppress proliferation and induce apoptosis of tumours. Tumour Biol 35, 5953–5963 (2014). 24682952 10.1007/s13277-014-1790-7

[b17] Yuan Y. *et al.* MicroRNA-203 inhibits cell proliferation by repressing DeltaNp63 expression in human esophageal squamous cell carcinoma. BMC cance 11, 57 (2011). 10.1186/1471-2407-11-57 PMC3044653 21299870

[b18] Takeshita N. *et al.* miR-203 inhibits the migration and invasion of esophageal squamous cell carcinoma by regulating LASP1. Int J Oncol 41, 1653–1661 (2012). 22940702 10.3892/ijo.2012.1614

[b19] Chen T. *et al.* MicroRNA-203 inhibits cellular proliferation and invasion by targeting Bmi1 in non-small cell lungcancer. Oncol Lett 9, 2639–2646 (2015). 26137120 10.3892/ol.2015.3080 PMC4473497

[b20] Zhang K. *et al.* miR-203 is a direct transcriptional target of E2F1 and causes G1 arrest in esophageal cancer cells. J Cell Physiol 230, 903–910 (2015). 25216463 10.1002/jcp.24821

[b21] Miao L. *et al.* miR-203 inhibits tumor cell migration and invasion via caveolin-1 in pancreatic cancer cells. Oncol Lett 7, 658–662 (2014). 24520289 10.3892/ol.2014.1807 PMC3919932

[b22] Hezova R. *et al.* Diagnostic and prognostic potential of miR-21, miR-29c, miR-148 and miR-203 in adenocarcinoma and squamous cell carcinoma of esophagus. Diagn Pathol 10, 42 (2015). 25928282 10.1186/s13000-015-0280-6 PMC4411933

[b23] Slaby O. *et al.* Dynamic changes in microRNA expression profiles reflect progression of Barrett’s esophagus toesophageal adenocarcinoma. Carcinogenesis 36, 521–527 (2015). 25784377 10.1093/carcin/bgv023

[b24] Stánitz É. *et al.* Alteration of miRNA expression correlates with lifestyle, social and environmental determinants inesophageal carcinoma. Anticancer Res 35, 1091–1097 (2015). 25667498

[b25] Lo Iacono. M. *et al.* p63 and p73 isoform expression in non-small cell lung cancer and corresponding morphological normal lung tissue. J Thorac Oncol 6, 473–481(2011). 21289519 10.1097/JTO.0b013e31820b86b0

[b26] Lo M. L. *et al.* p63 overexpression associates with poor prognosis in head and neck squamous cell carcinoma. Hum Pathol 36, 187–194 (2005). 15754296 10.1016/j.humpath.2004.12.003

[b27] Zhang J. *et al.* The protein levels of MCM7 and p63 in evaluating lesion severity of cervical disease. Int J Gynecol Cancer 23, 318–324 (2013). 23318911 10.1097/IGC.0b013e31827f6f06

[b28] Hu H. *et al.* Elevated expression of p63 protein in human esophageal squamous cell carcinomas. Int J Cancer 102, 580–583 (2002). 12447998 10.1002/ijc.10739

[b29] Takahashi Y. *et al.* Reduced expression of p63 has prognostic implications for patients with esophageal squamous cellcarcinoma. Oncol Rep 15, 323–328 (2006). 16391849

[b30] Cao L. Y., Yin Y., Li H., Jiang Y. & Zhang H. F. Expression and clinical significance of S100A2 and p63 in esophageal carcinoma. World J Gastroenterol 15, 4183–4188 (2009). 19725154 10.3748/wjg.15.4183 PMC2738816

[b31] Wang T. Y. *et al.* Histologic and immunophenotypic classification of cervical carcinomas by expression of the p53 homologue p63: a study of 250 cases. Hum Pathol 32, 479–486 (2001). 11381365 10.1053/hupa.2001.24324

[b32] Quade B. J. *et al.* Expression of the p53 homologue p63 in early cervical neoplasia. Gynecol Oncol 80, 24–29 (2001). 11136565 10.1006/gyno.2000.5953

[b33] Senoo M., Pinto F., Crum C. P. & McKeon F. p63 Is essential for the proliferative potential of stem cells in stratified epithelia. Cell 129, 523–536 (2007). 17482546 10.1016/j.cell.2007.02.045

[b34] Li Z. S. & Li Q. [The latest 2010 WHO classification of tumors of digestive system]. Zhonghua bing li xue za zhi Chinese journal of pathology 40, 351–354 (2011). 21756837

